# Book Review

**Published:** 1916-11

**Authors:** 


### BOOK REVIEWS

A PRACTICAL TREATISE ON DISORDERS OF THE SEXUAL FUNCTION IN THE MALE AND FEMALE—By Max Huhner, M. D., Chief of Clinic, Genitourinary Department, Mount Sinai Hospital Dispensary, New York City; Formerly, Attending Genitourinary Surgeon, Bellevue Hospital, Out-patient Department and Assistant Gynecologist, Mount Sinai Hospital Dispensary, New York City; Member, American Urological Association, American Medical Association, New York Urological Association, Fellow of the New York Academy of Medicine, etc.; Author, Sterility in the Male and Female and its Treatment, etc. F. A. Davis Co., Philadelphia, Publishers.

This is a very sensible book on sexual disorders and can be recommended heartily to those interested in the subject and few medical men can afford not to be interested in this common and important ailment. We must not expect every patient to come with the diagnosis already made. He may be the last one to suspect that his disturbance has any connection with his sexual organs, many of the symptoms being reflex and referred to other parts of his anatomy, or he may think them due to temperament—for instance the unusual or diminished sexual desire. He may be quite sure that there is no connection between his nervousness and irritability and the inflammation and irritation in his deep urethra and prostate.

Huhner is particularly fitted to discuss this subject because he has devoted most of his medical career to Genito-Urinary Diseases and neurology, and sexual disturbances are often dependent upon border-line conditions which appropriately fall within these two specialties.

The book is worth the price if it contained nothing more than the treatment of masturbation in the adult male.

